# Chitosan-Based Coacervate Polymers for Propolis Encapsulation: Release and Cytotoxicity Studies

**DOI:** 10.3390/ijms21124561

**Published:** 2020-06-26

**Authors:** Tabata Sato, Daphne Mello, Luana Vasconcellos, Artur J. M. Valente, Alexandre Borges

**Affiliations:** 1 Department of Dental Materials and Prosthodontics, Institute of Science and Technology, Sao Paulo State University, UNESP, Sao Paulo 12.245-700, Brazil; alexandre.borges@unesp.br; 2 Department of Bioscience and Buccal Diagnose, Institute of Science and Technology, Sao Paulo State University, UNESP, Sao Paulo 12.245-700, Brazil; daphne.cr.mello@unesp.br (D.M.); luana.marotta@unesp.br (L.V.); 3 Department of Chemistry, University of Coimbra, CQC, 3004-535 Coimbra, Portugal

**Keywords:** hydrogel, chitosan, pectin, DNA, propolis

## Abstract

Chitosan-DNA (CS-DNA) and Chitosan-Pectin (CS-P) hydrogels were formulated as a sustained drug delivery carrier for drug delivery. For this, hydrogels were prepared by emulsion technique: mixing aqueous phase of the CS and DNA or P solution with benzyl alcohol using a high-performance dispersing instrument. Green Propolis (GP) was incorporated by imbibition: hydrogels were placed in GP aqueous solution (70 µg/mL) for 2 h. The specimens were freeze-dried and then characterized using different techniques. In vitro cell viability and morphology were also performed using the MG63 cell line. The presence of P was evidenced by the occurrence of a strong band at 1745 cm^−1^, also occurring in the blend. DNA and CS-DNA showed a strong band at 1650 cm^−1^, slightly shifted from the chitosan band. The sorption of GP induced a significant modification of the gel surface morphology and some phase separation occurs between chitosan and DNA. Drug release kinetics in water and in saliva follow a two-step mechanism. Significant biocompatibility revealed that these hydrogels were non-toxic and provided acceptable support for cell survival. Thus, the hydrogel complexation of chitosan with DNA and with Pectin provides favorable micro-environment for cell growth and is a viable alternative drug delivery system for Green Propolis.

## 1. Introduction

Hydrogels are known to be crosslinked polymeric 3D networks capable of absorbing and retaining large amounts of water or biological fluids [1], having a microstructure similar to extracellular matrix (ECM) [2] and a biocompatibility with blood and biological tissues [3]. Additionally, hydrogels can be designed with tunable/responsive properties that make them highly sought after for biomedical and pharmaceutical (drug/gene deliver) applications [4,5].

Thus, many efforts have been directed towards hydrogel-based matrices for the development of vectors for the delivery of active substances for different targets. Among different strategies, the use of combinations of natural polymers [6] and biopolymers is mandatory. Following this approach, chitosan is a cationic biopolymer, obtained from the chitin deacetylation of crustaceous exoskeletons [7], showing antimicrobial biodegradable and biocompatible properties. Furthermore, due to the presence on its structure of amine and hydroxyl groups [8,9], the chitosan is highly chemically versatile, being used for several applications in pharmaceutical and medical areas [10], especially for wound healing applications [11], as they are similar to the natural extracellular matrix (ECM).

Chitosan hydrogels have wide applications, with no cytotoxic effects [12], such as local drug delivery [13] and tissue engineering [14], and their preparation context the dissolution of chitosan occurs in slightly acidic (pH < 6.5) aqueous solutions as a consequence of the protonation of its amine groups, allowing the formation of either chemical or physical crosslinked hydrogels [15]. Covalently crosslinked chitosan hydrogels are very effective in drug delivery systems [15,16]; however, they may show some drawbacks when used as, for example, toxic crosslinkers or initiators. The use of physical hydrogels makes it possible to overcome such limitations [17]. Among different approaches, and taking advantage of the positively charged chitosan, we decided to investigate the formation of CS-DNA and CS-P coacervate hydrogels. 

Deoxyribonucleic acid (DNA) is a biopolymer composed of deoxyribose sugar, able to carry genetic information [18]. DNA hydrogels have unique properties, such as biocompatibility, selective binding, and molecular recognition [19,20], creating a wide range of potential applications in the fields of drug delivery [4,21] and tissue engineering [22,23]. In fact, among biological polyelectrolytes, DNA has always attracted particular interest, and there have been numerous studies on the interactions between DNA and polycations [24,25,26]. The formation of coacervate hydrogels is achievable once DNA is completely ionized at neutral pH [27], enabling interaction with positively charged molecules such as chitosan [28].

Pectin (P) is another polianion at pH higher than ca. 9 that can form coacervate hydrogels with chitosan [29,30]. Pectin is a plant cell wall polysaccharide used as a gelling and stabilizing polymer in food or biomedical products due to its influence on human health, such as with respect to cholesterol levels and local bleeding [31,32]. 

Regarding the advancement of material design, studies on drug release kinetics are necessary to provide evidence of the newly developed systems [33]. Therefore, the present study aimed to design and fabricate Chitosan-DNA (CS-DNA) and Chitosan-Pectin (CS-P) matrices to elucidate the transport mechanism, which was tested as a sustained drug carrier for Green Propolis (GP), an herbal medicine produced by Apis mellifera honeybees, from Baccharis dracunculifolia DC (Asteraceae), and which is used as an anti-inflammatory [34], antifungal [35], and antioxidant drug [36] due to its high levels of phenolic acids [37].

## 2. Results and Discussion

### 2.1. Gel Characterization

**Figure 1:**
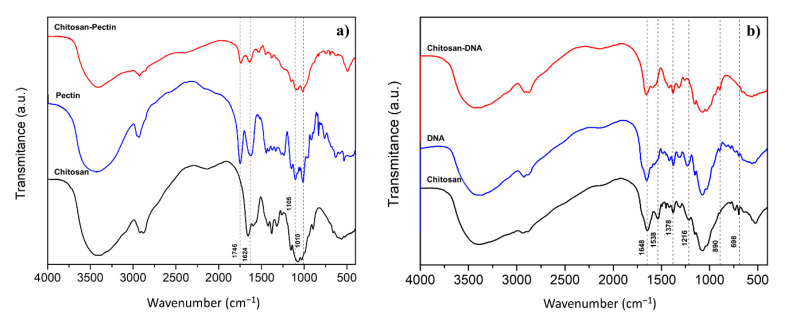
Infrared spectra of: (**a**) chitosan, pectin and the CS-P blend gel; and (**b**) chitosan, DNA and the CS-DNA blend gel.

Figure 1a,b shows the FTIR spectra of the CS-P and CS-DNA blend hydrogels, respectively, and the corresponding isolated polymers. The IR spectra of chitosan show two characteristic bands at 1648 cm^−1^ and 1538 cm^−1^ assigned to (C=O) stretching mode and N-H bending of amide group. A band at 1378 cm^−1^ can be attributed to the -CH3 symmetrical deformation and the strong band at 1076 cm^−1^ characterizes the C-O stretching [38,39]. Furthermore, the spectra show bands at 3417 cm^−1^ that indicate dimeric O-H stretching and at 1425 cm^−1^ indicating aromatic C-C stretching [40].

To confirm the occurrence of the coacervation process, the presence of pectin was evidenced by the occurrence of a strong band at 1745 cm^−1^, also occurring in the blend, ascribed to esterified groups, and at 1624 cm^−1^, assigned to free carboxyl groups [41]. The bands occurring at 1105 and 1010 cm^−1^, in the finger-print region, are typical of pectin polymers and can be attributed to the C=O stretching [30,42].

In the DNA and CS-DNA gels it is possible to observe a strong band at 1650 cm^−1^, slightly shifted from the chitosan band, assigned to the C2=O2 stretching vibration of cytosine [43]; the shoulder found at the blend and at DNA gels, at 1590 cm^−1^, can be attributed to a couple of C6=O6, C5-C6, and C4=C5 stretching vibrations of guanine [44]. The small modifications of the FTIR spectra around 1590 cm^−1^ may suggest a different base-pairing pattern of DNA alone and in the blend [43]. Further bands, in the CS-DNA spectrum, at 526 cm^−1^ and 890 cm^−1^, the latter only observed in the DNA-containing gels, indicate the deoxyribose region [45], and the deoxyribose-phosphate groups [46].

Excluding the T_max_ obtained at temperatures below 150 °C, caused by the evaporation of residual water, two main degradation steps were found at 234 °C and 292 °C for pectin [47,48] and chitosan [49], respectively. It is interesting that for the CS-P blend, two degradation steps can be found at 227 °C and a 548 °C; the latter can be justified by the degradation of chitosan. The former maximum degradation temperature, corresponding to ca. 63% weight loss, occurs at temperatures below the main transition steps for single pectin and chitosan. This can be justified by a decrease in the thermal stability of the blend as a consequence of ionic interactions between pectin and chitosan, leading to a plasticizer-like effect.

**Figure 2:**
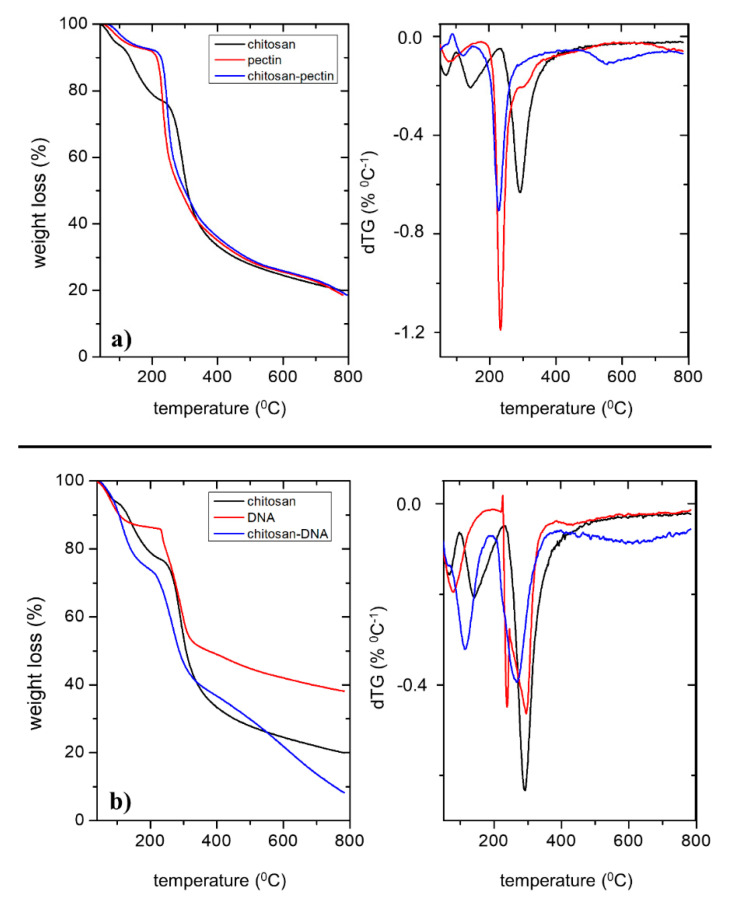
TGA and dTG curves of: (**a**) chitosan (^__^), pectin (^__^) and CS-P (^__^); and (**b**) chitosan (^__^), DNA (^__^) and CS-DNA (^__^).

The gels have also been characterized by thermogravimetric analysis (Figure 2a,b). In order to assess the main degradation temperatures, the maximum degradation rate was computed (dTG), and the corresponding temperature, T_max_, obtained.

The analysis shows that the first degradation step of DNA occurs at 238 °C [50], followed by a second degradation step at 295 °C, suggesting that the presence of different secondary structures might be present. However, the most remarkable difference between this blend, and the CS-P one, is that the main degradation step occurs at 268 °C, suggesting a higher degree of phase separation.

**Figure 3:**
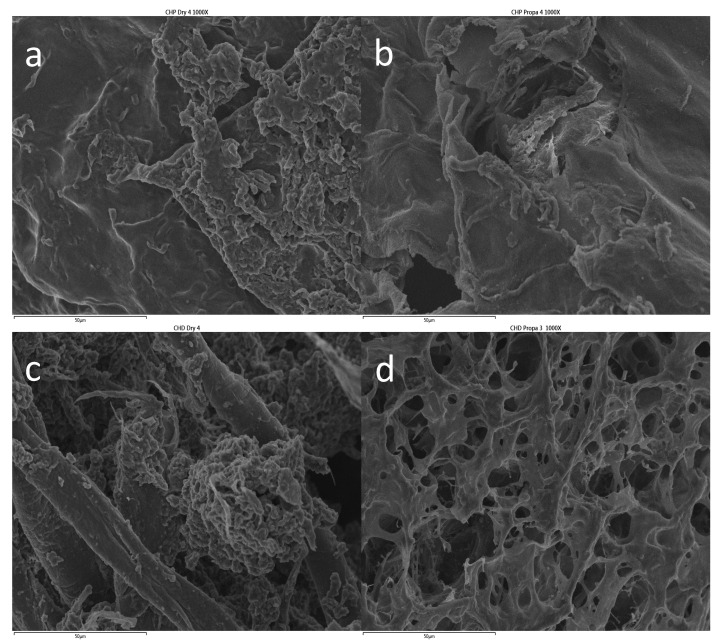
Scanning electron micrographs of CS-P (**a**,**b**) and CS-DNA (**c**,**d**) before (**a**,**c**) and after (**b**,**d**) GP loading. ×1000 magnification. Scale = 50 µm.

From the analysis of the surface morphology of CS-P and CS-DNA gels (Figure 3), it can be observed that the surface of the latter shows a more heterogeneous structure, characterized by well-defined aggregates and fiber-like structures. On the other hand, for CS-P, the surface morphology is a more featureless, non-porous structure, indicating some irregularities that might be related to some phase separation. This morphology can increase stability by providing higher resistance [51]. However, after the GP sorption, a significant change in the surface morphology of CS-DNA is observed, i.e., the surface becomes highly porous. This has been reported for other DNA-containing composites, such as PVA-DNA [43], and suggests that the GP has a significant effect or even interaction with the polymers. This might also justify the lowest amounts of GP release by CS-DNA (see the discussion below), when compared with the CS-P, despite its higher porosity. However, the surface of CS-P does not undergo relevant modification upon GP loading, indicating that the pectin is playing a major role [52].

### 2.2. Loading and Drug Release

A wide variety of polymer-based materials have been developed for drug delivery, such as biodegradable chitosan hydrogels [53]. Table 1 shows the encapsulation efficiency of GP into CS-P and CS-DNA blend gels. 

It can be seen that the EE in the CS-DNA is significantly lower (ca. 3.5-fold lower) than those obtained for CS-P. This might be related to the occurrence of higher phase separation in the CS-DNA gel and also the lower swelling degree of this gel (Q = 700%) when compared to that of CS-P (Q = 1109%). However, it is surprising that the cumulative release, in percentage, is higher for the CS-DNA gel. This might be due to the change in the structure of the pectin-DNA gel, which becomes a more porous surface structure. In any case, the absolute value of the maximum released amount of GP is always higher for CS-P.

**Figure 4:**
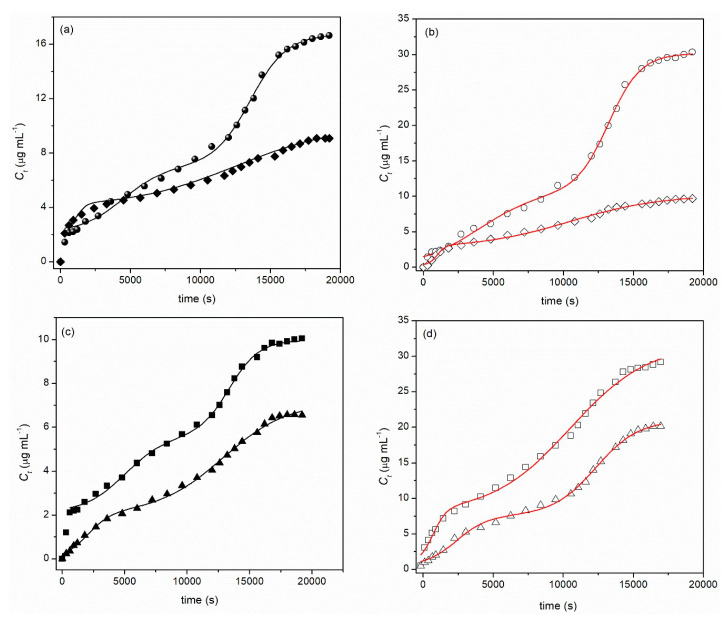
Release profiles of Green Propolis from: (**a,b**) CS-P gels to water (diamonds) and simulated saliva (circles), at 25 °C (**a**) and 37 °C (**b**); (**c,d**) CS-DNA gels to water (triangles) and simulated saliva (squares), at 25 °C (**c**) and 37 °C (**d**)). Solid lines were obtained by fitting Equation (1) to the experimental data.

Figure 4 shows the release kinetics of GP from CS-P gels to water (diamond) and simulated saliva (spheres), at 25 and 37 °C (black and white data points, respectively; Figure 4a,b). The solid lines were obtained by fitting Equation (1) to the experimental data. Figure 4c,d shows the release kinetics of GP from CS-DNA gels to water (triangle) and simulated saliva (square), at 25 and 37 °C (black and white data points, respectively). Solid lines were obtained by fitting Equation (1) to the experimental data.

As a first approach, we can observe that in all cases, the release kinetics follows a two-step mechanism; however, it is also clear that such a mechanism occurs concomitantly when no plateau is observed just before the beginning of the second significant release—this will be discussed later. Two other points are worth noticing: the cumulative release increase with increasing temperature (the exception occurs for the release from CS-P to water), which is more significant in the case of the CS-P gel; and the boost release that can be observed during the first 10 min of release at 25 °C. The latter process might be justified by a surface phenomenon (either surface erosion or preferential adsorption) [54]; however, that process is somewhat hindered by increasing the temperature and, consequently, the origin of mass transport ceases to be relevant.

To obtain better insight into the release mechanism, several heuristic models were tested (e.g., Weibull, Logistic, Korsmeyer-Peppas and Peppas-Sahlin [21,55,56] without success—results not shown. However, assuming that the release is characterized by a biphasic release that may occur simultaneously, as can be inferred from the continuous increase of the GP as a function of time (i.e., no two plateaus of cumulative release concentration can be observed as a function of time), the following equation was used [57]:

(1) $$C_{t}=C_{0}+\left(C_{eq}-C_{0}\right)\left(\frac{p}{1+10^{\left(logt_{1}-t\right)k_{1}}}+\frac{p}{1+10^{\left(logt_{2}-t\right)k_{2}}}\right)$$

where *C*_0_, *C_t_* and *C_eq_* are the initial, cumulative and equilibrium release concentrations, respectively, of GP, logt1 and *logt*_2_ correspond to the first and second EC_50_ (i.e., the concentrations of the drug that give half-life response), and p is the contribution of each phase for the whole release profile. All the fitting parameters, summarized in Table 2, were estimated on the basis of a least-squares method, with a confidence degree of 95%, by using Origin 8.5 software, while keeping *C*_0_ as a constant equal to zero.

From the analysis of Figure 4a,b and *C*_*eq*_ summarized in Table 2, it can also be concluded that the amount of GP released is significantly higher in synthetic saliva than in water. The amount of GP released reaches a value between 14% and 36% of the encapsulated amount of GP. This may be attributed to the effect of ionic strength on these gels. Once both gels are ampholytic gels, an increase in the ionic strength may lead to a swelling effect, which may stabilize both polymers [58,59], thus contributing to the enhancement of GP release. This is also followed by a significant increase in the EC_50_ for the first step release (*logt*_1_) compared to those observed for the water system. Finally, concerning the contribution of both steps for the whole release process, the first step mechanism contributes ca. 30% for the total release of GP for all systems, suggesting that the main release process is probably affected by some polymeric relaxation processes, further facilitating the mobility and release of the GP [60,61,62].

### 2.3. Biological Analysis

**Figure 5:**
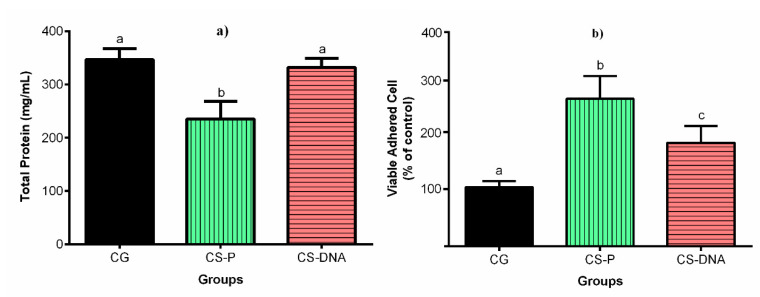
Mean ± SD for in vitro tests for (**a**) measurement of cell viability MTT assay and (**b**) total protein production. Values that do not share the same superscript letters are significantly different from each other (*p* < 0.05).

The results of cell viability MTT assay and total protein production are shown in Figure 5.

With respect to total protein (Figure 5b), there was no statistical difference between the control group and CS-DNA (*p* > 0.001), but the values from the CS-P experimental group were lower than those for the control group, with statistical significance (*p* = 0.0002). 

The cell viability was obtained by MTT assay, and measurements of cell viability of experimental groups were compared to the control group (100%) (Figure 5a). The CS-P and CS-DNA were positive influences in the cell culture and improved the number of viable cells. However, the CS-P experimental group exhibited a statistically significant difference with respect to the control group (*p* < 0.0001), which presented lower cell viability. This result is in accordance with previous studies, which observed an increase of viable cells in different linage cells when chitosan was used as a biomaterial base [63,64,65]. The rates of cell viability exhibited by both groups make them suitable for biomedical use according to the International Organization for Standardization (ISO) and the national standards for Biological Evaluation of Medical Devices, ISO 10993-5 [66]. 

**Figure 6:**
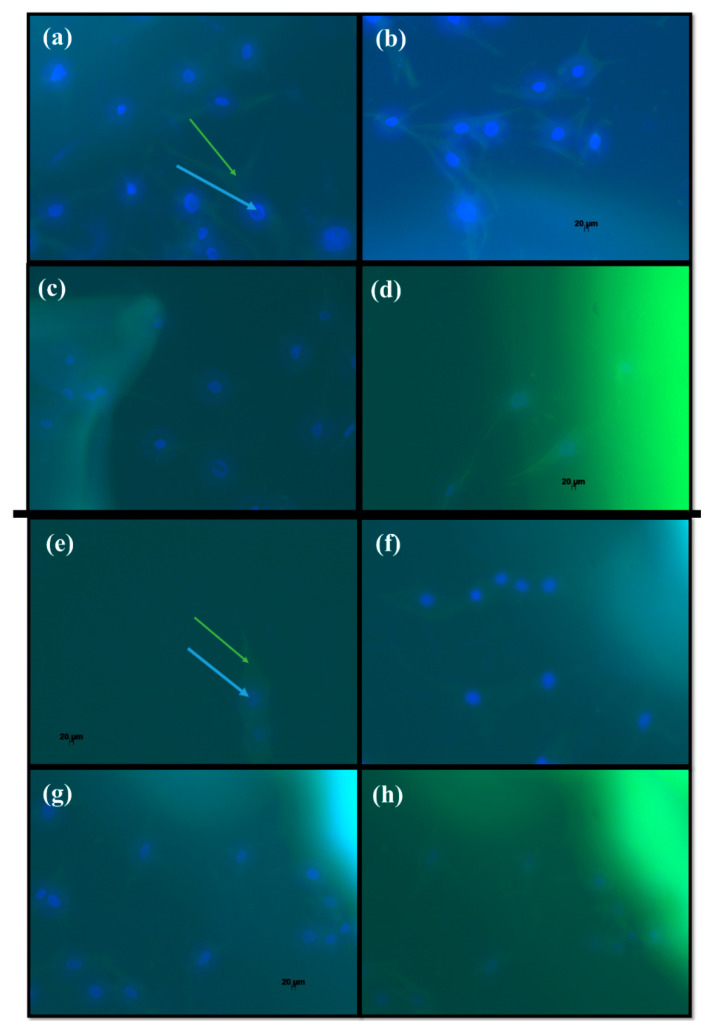
Fluorescence cell imaging: Alexa Fluor 488 conjugated to phalloidin and DAPI targeting actin (green arrow) and nuclear DNA (blue arrow) for cell-CS-DNA (**a**–**d**) and CS-P (**e**–**h**) contact, respectively.

Figure 6 shows fluorescence microscopic visualization of cells in contact with hydrogel.

Demonstrating the labeled cellular targets of the hydrogels, the images reveal cell-to-cell (Figure 6a,b,e,f), cell-CS-DNA (Figure 6c,d) and cell-CS-P (Figure 6g,h) contact. These fluorescence microscopic images illustrate the cell distribution on the hydrogel surfaces to be dependent on their migration and proliferation, as distinct from the homogeneous distribution that occurs in process in which the cells are encapsulated inside the gel [67].

## 3. Materials and Methods 

### 3.1. Materials

Chitosan with an acetylation degree of 15 mol% (Mw: 87 × 103 g mol^−1^) was purchased from Golden-Shell Biochemical (Yuhuan, China). Pectin from citrus peel and double-stranded (ds) DNA (sodium salt from salmon testes), with a polymerization average degree of ca. 2000 base pairs, were purchased from Sigma. Benzyl alcohol (99%) was obtained from Merck KGaA (Darmstadt, Germany).

Aqueous extract of Green Propolis was obtained from Apis Flora (Ribeirão Preto, SP, Brazil). Millipore-Q water was used to prepare the solutions.

Simulated saliva was made by Indiana University modified method [68], prepared according to Fusayama et al. (1963) [69].

### 3.2. Hydrogel Preparation

Both cocervate hydrogels (CS-DNA and CS-P) were prepared using the emulsion technique (oil-in-water). The preparation of the former gel was done using an acetate buffer (pH 6). This buffer was chosen to balance the cationic density of chitosan and the chemical instability of DNA [70]. 

Thus, an aqueous chitosan solution (1% *w*/*v*) was initially prepared in acetate buffer and then filtered through a paper filter to remove insoluble substances [30]. DNA and pectin aqueous solutions were prepared in phosphate buffer at pH 6 and pH 9.2, respectively, under stirring for 12 h, at room temperature. The emulsions were obtained by mixing aqueous phase of the Ch solution (1 mL) and DNA solution (1 mL) (1:1 (*v*/*v*) ratio of Ch:DNA) or Pectin solution (1 mL) (1:1 (*v*/*v*) ratio of Ch:Pectin) with benzyl alcohol (5 mL) using a Ultra-Turrax at 31-34000 rpm min-1 for 5 min [71].

### 3.3. Hydrogel Characterization

The synthesized hydrogels were freeze-dried prior to their characterization using various techniques. Attenuated reflection infrared spectroscopy (ATR-FTIR) was carried out in a Cary 630 FTIR Spectrometer (Agilent, Santa Clara, CA, USA), with wavenumber ranging from 650 cm^−1^ to 4000 cm^−1^.

The thermogravimetric analysis (TGA) was performed in a thermogravimetric analyzer (TG209 F3 Tarsus Netzsch Instruments, NETZSCH-Gerätebau GmbH, Selb, Germany). Samples of ca. 5 mg were weighed in alumina pans and heated from 25 °C to 800 °C at a 10 °C min^−1^ rate, under an N2 atmosphere (20 mL min^−1^).

The surface morphologies of hydrogels were analyzed using scanning electron microscopy (SEM, JEOL USA, Inc., Peabody, MA, USA) with a JEOL model 5310 scanning microscope (JEOL USA, Inc., Peabody, MA, USA) operating under low vacuum at 15 kV. The membranes were submitted to a fast cryogenic treatment by diving gel samples into liquid nitrogen for 10 s, and then they were left overnight in a freeze dryer (Free Zone 4.5-Labconco) before being coated with a gold film.

The effect of water and simulated fluids on hydrogels before and after the loading of Green Propolis was assessed by swelling degree (*Q*). Different samples of the same membrane were cut, weighed, and immersed in water or simulated solutions and left to reach swelling equilibrium (2 days). After this time, membranes were removed from the solution, any drops of solution were wiped off, and then the weight of the membrane was measured using an analytical balance ADA 120LE (Sartorius AG, Göttingen, Germany). Each experiment was repeated twice. The swelling degree of the gel (*Q*) was calculated by using the following equation:

(2) $$Q=m_{w}/m_{x}$$

where *m_w_* and *m_x_* are the masses of water (or solution) in the swollen gel and in the xerogel, respectively. The mass of the xerogel samples was obtained using the weight of the sample after synthesis and taking into account the solid content of the gel [43].

### 3.4. Loading and Release of Green Propolis

Green Propolis was incorporated into the coacervate gels by imbibition. A 70 µg/mL GP aqueous solution was prepared; after that, hydrogel samples (of ca. 20 mg) were placed in that volume of GP solution and left there for 2 h, at 25 °C, using a thermostatic bath.

The encapsulation (EE) efficiency was calculated by using, respectively, the following equations:

(3) $$EE=\frac{\left(total\;amount\;of\;GP-nonbound\;GP\right)}{total\;amount\;of\;GP}\times 100$$

To determine the GP release kinetics, the hydrogel samples, after GP encapsulation, were placed in vessels containing 20 mL of water, and kept in an incubator (Labwit, ZWY-100H, Burwood East, VIC, USA), at 37 °C and 150 rpm. At predetermined time intervals (20 min), aliquots of the supernatant were taken from the vessel and stored for GP quantification. Meanwhile, that volume was replaced with water. The process was repeated until release equilibrium was attained. The GP released into the supernatant was quantified spectrophotometrically by measuring the absorbance in a range of 200 to 800 nm using a double-beam Shimadzu UV-2100 spectrometer (SHIMADZU EUROPA, Duisburg, Germany).

### 3.5. Biological Analysis

In this present study, an established lineage of osteoblast-like cells (MG63) acquired from Rio de Janeiro Cell Bank (Rio de Janeiro, RJ, Brazil) was used. Cells were cultured in Modified Eagle’s medium (Life Technologies, NY, USA) containing 10% fetal bovine serum (Life Technologies, NY, USA) and penicillin (100 U/mL) at 37 °C in a humidified atmosphere of 5% CO_2_. The culture medium was changed every two days until the moment test. All tests were performed according to ISO 10993 guidelines, with three independent experiments.

Initially, five samples for each material were placed into 24-well plates (TPP, Curitiba, Brazil), with 2 × 104 cells subsequently being placed in each well. After 7 days for culture, an MTT colorimetric assay was performed with incubation in 3-(4,5-dimethylthiazol-2-yl)-2,5-diphenyltetrazolium bromide (Sigma-Aldrich, St Louis, MO, USA); then, the cells were lysed with a propanol acid solution (Sigma-Aldrich, St Louis, MO, USA) for colorimetric measurement in a spectrophotometer (EL 808 BioTek Instruments, Winooski, VT, USA) at 570 nm, as previously described [72]. The values were expressed as percentage of viable cells compared to the control group (only cells), which was considered to be 100%.

Proteins were extracted with 0.1% sodium dodecyl sulfate (Sigma-Aldrich St Louis MO, USA), followed by addition of Lowry solution (Sigma-Aldrich, St Louis, MO, USA). The extract was diluted in Folin–Ciocalteu reagent (Sigma-Aldrich, St Louis, MO, USA) and the absorbance was assessed in a spectrophotometer (8582 Micronal, São Paulo, Brazil) at 680 nm [72]. The total protein content was calculated from a bovine serum albumin standard and expressed in μg/mL.

Finally, for cell morphology of the actin cytoskeleton and cell nucleus adhered, the cultures grown on the samples for 3 days and were fixed for 15 min using 4% paraformaldehyde (Synth, São Paulo, Brasil) in 0.1M sodium phosphate buffer (PBS) (pH 7.2), at room temperature. Briefly, they were permeabilized with 0.5% Triton X-100 (Sigma Aldrich, St Louis, MO, USA) in PB for 15 min, followed by 1% ovalbumin for 30 min, and Alexa Fluor 488 Phalloidin (Invitrogen Eugene, Oregon, USA) was subsequently added for 20 min, followed by two washes with PBS [73]. The analyses were performed on the well cells that were in contact with the samples under the fluorescence microscope (Microscope Carl Zeiss Microlimaging GmbH—Axiovert 40C, Germany). At the end of the procedure, Fluoroshield with DAPI (Sigma-Aldrich, St Louis, USA) was added. The digital images were visualized using the Axio Vision 7.0 software.

## 4. Conclusions

This study reports a necessary bridge between chemistry and biology, since the control of properties is imperative for in vivo applications of new biomaterials. To achieve this, biocompatible coacervate hydrogels based on chitosan, with either salmon DNA and pectin, were synthesized by the emulsion technique for drug delivery of the herbal medicine Green Propolis. In addition to a chemical and morphological characterization, the release transport of GP that had been previously encapsulated into gels in different media and at different temperatures were assessed along with the gel’s cytotoxicity.

The hydrogel release kinetics, analyzed at different temperatures (37 °C and 25 °C) and with different vehicles (water and saliva), exhibited a two-step mechanism, a cumulative release that increased proportionally to the temperature, significantly for the CS-P, with a boost release at 25 °C. Moreover, the amount of GP released was higher in saliva than in water. 

Regarding cell viability, both hydrogels positively influenced the cell culture and improved the number of viable cells. CS-DNA showed classical growth of total protein assay, statistically similar to the control group and different from CS-P.

Considering these factors, this study reports the successful fabrication of hydrogels composed of chitosan-DNA and chitosan-pectin, potential alternatives to drug delivery systems, and preliminary evidence suggesting that both proposed matrices are favorable micro-environments for potential use as materials in the biomedical field. 

## Figures and Tables

**Table 1:** Encapsulation efficiency and cumulative release of GP in CS-P and CS-DNA coacervate gels, at 25 °C.

	CS-P		CS-DNA	
	25 °C	37 °C	25 °C	37 °C
	**Water**			
***EE* (%)**	19	21	7	8
**Cumulative release (%)**	13.2	12.1	25.7	30
	**Simulated Saliva**			
***EE* (%)**	30	38	8	10
**Cumulative release (%)**	13.9	19.9	31.6	36.5

**Table 2:** Fitting parameters of Equation (1) to experimental release data of GP loaded into CS-P and CS-DNA gels to simulated saliva, at 25 °C and 37 °C.

	CS-P		CS-DNA	
	25 °C	37 °C	25 °C	37 °C
	**Water**			
*C_eq_*/(μg mL^−1^)	10.1 (±0.4)	10.2 (±0.3)	7.2 (±0.2)	9.6 (±0.2)
log*t_1_*/s	1051 (±73)	1005 (±74)	2051 (±203)	2851 (±214)
log*t_2_*/s	12,571 (±390)	10,460 (±360)	12,878 (±242)	14,005 (±158)
*k_1_*/s^−1^	1.2 (±0.3) × 10^−3^	1.5 (±0.5) × 10^−3^	4.7 (±0.2) × 10^−4^	3.5 (±0.4) × 10^−4^
*k_2_*/s^−1^	1.2 (±0.2) × 10^−4^	1.3 (±0.2) × 10^−4^	1.7 (±0.2) × 10^−4^	2.5 (±0.2) × 10^−4^
*P*	0.24 (±0.04)	0.26 (±0.04)	0.29 (±0.04)	0.34 (±0.02)
R^2^	0.9963	0.9952	0.9970	0.9972
	**Simulated Saliva**			
*C_eq_*/(μg mL^−1^)	16.7 (±0.2)	30.2 (±0.3)	10.1 (±0.6)	14.6 (±0.6)
log*t_1_*/s	4468 (±363)	3948 (±529)	4906 (±300)	969 (±127)
log*t_2_*/s	13,567 (±131)	13,225 (±118)	13,379 (±147)	11,972 (±321)
*k_1_*/s^−1^	2.9 (±0.4) × 10^−4^	2.0 (±0.3) × 10^−4^	2.7 (±0.3) × 10^−4^	8 (±2) × 10^−4^
*k_2_*/s^−1^	3.3 (±0.3) × 10^−4^	3.8 (±0.4) × 10^−4^	3.3 (±0.4) × 10^−4^	1.4 (±0.2) × 10^−4^
*p*	0.34 (±0.03)	0.36 (±0.03)	0.43 (±0.04)	0.24 (±0.04)
R^2^	0.9974	0.9978	0.9981	0.9951

The values inside parenthesis are the standard deviations.

## Abbreviations

CS	Chitosan
P	Pectin
DNA	Deoxyribonucleic acid
GP	Green propolis
TGA	Thermogravimetric analysis
dTG	Derivative thermogravimetry
PVA	Polyvinyl alcohol
MTT	3-(4,5-Dimethylthiazol-2-Yl)-2,5-Diphenyltetrazolium Bromide

## References

[1] Peppas N.A. (1986). Hydrogels in Medicine and Pharmacy: Fundamentals.

[2] Ma S., Yu B., Pei X., Zhou F. (2016). Structural hydrogels. Polymer.

[3] Gong J.P. (2006). Friction and lubrication of hydrogels—Its richness and complexity. Soft Matter.

[4] Costa D., Albuquerque T., Queiroz J.A., Valente A.J.M. (2019). A co-delivery platform based on plasmid DNA peptide-surfactant complexes: Formation, characterization and release behavior. Colloids Surf B Biointerfaces.

[5] Croisfelt F.M., Tundisi L.L., Ataide J.A., Silveira E., Tambourgi E.B., Jozala A.F., Souto E.M.B., Mazzola P.G. (2019). Modified-release topical hydrogels: A ten-year review. J. Mater. Sci..

[6] Agnihotri S., Singhal R. (2018). Synthesis and characterization of novel poly (acrylic acid/sodium alginate/sodium humate) superabsorbent hydrogels. Part II: The effect of SH variation on Cu 2+, Pb 2+, Fe 2+ metal ions, MB, CV dye adsorption study. J. Polym. Environ..

[7] Qin X., Zhang H., Wang Z., Jin Y. (2018). Magnetic chitosan/graphene oxide composite loaded with novel photosensitizer for enhanced photodynamic therapy. RSC Adv..

[8] Kyzas G.Z., Bikiaris D.N. (2015). Recent modifications of chitosan for adsorption applications: A critical and systematic review. Mar. Drugs.

[9] Martins A.F., Bueno P.V., Almeida E.A., Rodrigues F.H., Rubira A.F., Muniz E.C. (2013). Characterization of N-trimethyl chitosan/alginate complexes and curcumin release. Int. J. Biol. Macromol..

[10] Zhao D., Yu S., Sun B., Gao S., Guo S., Zhao K. (2018). Biomedical applications of chitosan and its derivative nanoparticles. Polymers.

[11] Chien K.B., Chung E.J., Shah R.N. (2014). Investigation of soy protein hydrogels for biomedical applications: Materials characterization, drug release, and biocompatibility. J. Biomater. Appl..

[12] Pakzad Y., Ganji F. (2016). Thermosensitive hydrogel for periodontal application: In vitro drug release, antibacterial activity and toxicity evaluation. J. Biomater. Appl..

[13] Jiang Z., Han B., Liu W., Peng Y. (2017). Evaluation on biological compatibility of carboxymethyl chitosan as biomaterials for antitumor drug delivery. J. Biomater. Appl..

[14] Zhou H.Y., Jiang L.J., Cao P.P., Li J.B., Chen X.G. (2015). Glycerophosphate-based chitosan thermosensitive hydrogels and their biomedical applications. Carbohydr. Polym..

[15] Berger J., Reist M., Mayer J.M., Felt O., Peppas N., Gurny R. (2004). Structure and interactions in covalently and ionically crosslinked chitosan hydrogels for biomedical applications. Eur. J. Pharm. Sci..

[16] Aydınoğlu D., Ünal M. (2019). Evaluation of the influence of spirulina microalgae on the drug delivery characteristics of genipin cross-linked chitosan hydrogels. Int. J. Polym. Mater..

[17] Reyna-Urrutia V.A., Mata-Haro V., Cauich-Rodriguez J.V., Herrera-Kao W.A., Cervantes-Uc J.M. (2019). Effect of two crosslinking methods on the physicochemical and biological properties of the collagen-chitosan scaffolds. Eur. Polym. J..

[18] Uzumcu A.T., Guney O., Okay O. (2016). Nanocomposite DNA hydrogels with temperature sensitivity. Polymer.

[19] Murakami Y., Maeda M. (2005). DNA-responsive hydrogels that can shrink or swell. Biomacromolecules.

[20] Ishizuka N., Hashimoto Y., Matsuo Y., Ijiro K. (2006). Highly expansive DNA hydrogel films prepared with photocrosslinkable poly (vinyl alcohol). Colloids. Surf. A Physicochem. Eng. Asp..

[21] Costa D., Valente A.J., Miguel M.G., Queiroz J. (2011). Gel network photodisruption: A new strategy for the codelivery of plasmid DNA and drugs. Langmuir.

[22] Um S.H., Lee J.B., Park N., Kwon S.Y., Umbach C.C., Luo D. (2006). Enzyme-catalysed assembly of DNA hydrogel. Nat. Mater..

[23] Costa D., Valente A.J.M., Miguel M.G., Queiroz J. (2014). Plasmid DNA hydrogels for biomedical applications. Adv. Colloid. Interfac..

[24] Costa D., Valente A.J., Queiroz J. (2015). Stimuli-responsive polyamine-DNA blend nanogels for co-delivery in cancer therapy. Colloids Surf. B Biointerfaces.

[25] Jorge A.F., Dias R.S., Pereira J.C., Pais A.A.C.C. (2010). DNA Condensation by pH-Responsive Polycations. Biomacromolecules.

[26] Jorge A.F., Morán M.C., Vinardell M.P., Pereira J.C., Dias R.S., Pais A.A. (2013). Ternary complexes DNA–polyethylenimine–Fe (iii) with linear and branched polycations: Implications on condensation, size, charge and in vitro biocompatibility. Soft Matter..

[27] Bravo-Anaya L.M., Rinaudo M., Martínez F.A.S. (2016). Conformation and rheological properties of calf-thymus DNA in solution. Polymers.

[28] Bravo-Anaya L.M., Soltero J.F.A., Rinaudo M. (2016). DNA/chitosan electrostatic complex. Int. J. Biol. Macromol..

[29] Neufeld L., Bianco-Peled H. (2017). Pectin–chitosan physical hydrogels as potential drug delivery vehicles. Int. J. Biol. Macromol..

[30] Cesar Filho M., Bueno P.V., Matsushita A.F., Rubira A.F., Muniz E.C., Durães L., Murtinho D.M., Valente A.J. (2018). Synthesis, characterization and sorption studies of aromatic compounds by hydrogels of chitosan blended with β-cyclodextrin-and PVA-functionalized pectin. RSC Adv..

[31] Mohnen D. (2008). Pectin structure and biosynthesis. Curr. Opin. Plant Biol..

[32] Thakur B.R., Singh R.K., Handa A.K., Rao M. (1997). Chemistry and uses of pectin—a review. Crit. Rev. Food Sci. Nutr..

[33] Fu Y., Kao W.J. (2010). Drug release kinetics and transport mechanisms of non-degradable and degradable polymeric delivery systems. Expert Opin. Drug Deliv..

[34] De Barros M.P., Sousa J.P.B., Bastos J.K., de Andrade S.F. (2007). Effect of Brazilian green propolis on experimental gastric ulcers in rats. J. Ethnopharmacol..

[35] Santos V., Pimenta F., Aguiar M., Do Carmo M., Naves M., Mesquita R. (2005). Oral candidiasis treatment with Brazilian ethanol propolis extract. Phytother. Res..

[36] Nakajima Y., Shimazawa M., Mishima S., Hara H. (2007). Water extract of propolis and its main constituents, caffeoylquinic acid derivatives, exert neuroprotective effects via antioxidant actions. Life Sci..

[37] Bankova V.S., de Castro S.L., Marcucci M.C. (2000). Propolis: Recent advances in chemistry and plant origin. Apidologie.

[38] Fernandes Queiroz M., Melo K.R., Sabry D.A., Sassaki G.L., Rocha H.A. (2014). Does the use of chitosan contribute to oxalate kidney stone formation?. Mar. Drugs.

[39] Souza N.L.G.D., Salles T.F., Brandão H.M., Edwards H.G.M., de Oliveira L.F.C. (2015). Synthesis, Vibrational Spectroscopic and Thermal Properties of Oxocarbon Cross Linked Chitosan. J. Braz. Chem. Soc..

[40] Krishnaveni B., Ragunathan R. (2015). Extraction and characterization of chitin and chitosan from F. solani CBNR BKRR, Synthesis of their bionanocomposites and study of their productive application. Int. J. Pharm. Sci. Rev. Res..

[41] Gnanasambandam R., Proctor A. (2000). Determination of pectin degree of esterification by diffuse reflectance Fourier transform infrared spectroscopy. Food Chem..

[42] Silverstein R.M., Bassler G.C. (1962). Spectrometric identification of organic compounds. J. Chem. Educ..

[43] Papancea A., Valente A.J., Patachia S., Miguel M.G., Lindman B. (2008). PVA− DNA cryogel membranes: Characterization, swelling, and transport studies. Langmuir.

[44] Lindqvist M., Gräslund A. (2001). An FTIR and CD study of the structural effects of G-tract length and sequence context on DNA conformation in solution. J. Mol. Biol..

[45] Ede S.R., Ramadoss A., Anantharaj S., Nithiyanantham U., Kundu S. (2014). Enhanced catalytic and supercapacitor activities of DNA encapsulated β-MnO 2 nanomaterials. Phys. Chem. Chem. Phys..

[46] Froehlich E., Mandeville J., Weinert C., Kreplak L., Tajmir-Riahi H. (2010). Bundling and aggregation of DNA by cationic dendrimers. Biomacromolecules.

[47] Rachini A., Le Troedec M., Peyratout C., Smith A. (2009). Comparison of the thermal degradation of natural, alkali-treated and silane-treated hemp fibers under air and an inert atmosphere. J. Appl. Polym. Sci..

[48] Liu Y., Sun Y., Ding G., Geng Q., Zhu J., Guo M., Duan Y., Wang B., Cao Y. (2015). Synthesis, characterization, and application of microbe-triggered controlled-release kasugamycin–pectin conjugate. J. Agric. Food Chem..

[49] Corazzari I., Nisticò R., Turci F., Faga M.G., Franzoso F., Tabasso S., Magnacca G. (2015). Advanced physico-chemical characterization of chitosan by means of TGA coupled on-line with FTIR and GCMS: Thermal degradation and water adsorption capacity. Polym. Degrad. Stab..

[50] Safaee M.M., Gravely M., Lamothe A., McSweeney M., Roxbury D. (2019). Enhancing the Thermal Stability of Carbon Nanomaterials with DNA. Sci. Rep..

[51] Stealey S., Guo X., Ren L., Bryant E., Kaltchev M., Chen J., Kumpaty S., Hua X., Zhang W. (2019). Stability improvement and characterization of bioprinted pectin-based scaffold. J. Appl. Biomater. Func..

[52] Cazorla-Luna R., Notario-Pérez F., Martín-Illana A., Ruiz-Caro R., Tamayo A., Rubio J., Veiga M.D. (2019). Chitosan-based mucoadhesive vaginal tablets for controlled release of the Anti-HIV drug tenofovir. Pharmaceutics.

[53] Varaprasad K., Vimala K., Ravindra S., Reddy N.N., Reddy G.S.M., Raju K.M. (2012). Biodegradable chitosan hydrogels for in vitro drug release studies of 5-flurouracil an anticancer drug. J. Polym. Environ..

[54] Macha I.J., Ben-Nissan B., Vilchevskaya E.N., Abali B.E., Mueller W.H., Rickert W. (2019). Drug delivery from polymer-based nanopharmaceuticals—An experimental study complemented by a simulation of selected diffusion processes. Front. Bioeng. Biotechnol..

[55] Issa M.G., Pessole L., Takahashi A.I., Andréo Filho N., Ferraz H.G. (2012). Physicochemical and dissolution profile characterization of pellets containing different binders obtained by the extrusion-spheronization process. Braz. J. Pharm. Sci..

[56] Peppas N.A., Sahlin J.J. (1989). A simple equation for the description of solute release. III. Coupling of diffusion and relaxation. Int. J. Pharm..

[57] Liu L., Hu S., Wang Y., Yang S., Qu J. (2018). Optimizing the synthesis of core/shell structure Au@ Cu 2 S nanocrystals as contrast-enhanced for bioimaging detection. Sci. Rep..

[58] Baker J.P., Stephens D.R., Blanch H.W., Prausnitz J.M. (1992). Swelling equilibria for acrylamide-based polyampholyte hydrogels. Macromolecules.

[59] Valente A.J., Cruz S.M., Murtinho D.M., Miguel M.G., Muniz E.C. (2013). DNA–poly (vinyl alcohol) gel matrices: Release properties are strongly dependent on electrolytes and cationic surfactants. Colloids Surf. B. Biointerfaces.

[60] Siepmann J., Podual K., Sriwongjanya M., Peppas N., Bodmeier R. (1999). A new model describing the swelling and drug release kinetics from hydroxypropyl methylcellulose tablets. J. Pharm. Sci..

[61] Faisant N., Siepmann J., Benoit J. (2002). PLGA-based microparticles: Elucidation of mechanisms and a new, simple mathematical model quantifying drug release. Eur. J. Pharm. Sci..

[62] Polishchuk A.Y., Zaikov G.E. (1997). Multicomponent transport in polymer systems for controlled release.

[63] Dinescu S., Ionita M., Ignat S.-R., Costache M., Hermenean A. (2019). Graphene oxide enhances chitosan-Based 3D scaffold properties for bone tissue engineering. Int. J. Mol. Sci..

[64] Li Y., Qiao Z., Yu F., Hu H., Huang Y., Xiang Q. (2019). Transforming growth factor-beta3/chitosan sponge (TGF-beta3/CS) facilitates osteogenic differentiation of human periodontal ligament stem cells. Int. J. Mol. Sci..

[65] Singh Y.P., Dasgupta S., Bhaskar R. (2019). Preparation, characterization and bioactivities of nano anhydrous calcium phosphate added gelatin–chitosan scaffolds for bone tissue engineering. J. Biomater. Sci. Polym. Ed..

[66] Normalizacyjny P.P.K. (2009). Biological Evaluation of Medical Devices—Part 5: Tests for In Vitro Cytotoxicity (ISO 10993-5:2009).

[67] Hwang C.M., Sant S., Masaeli M., Kachouie N.N., Zamanian B., Lee S.-H., Khademhosseini A. (2010). Fabrication of three-dimensional porous cell-laden hydrogel for tissue engineering. Biofabrication.

[68] Swartz M.L., Phillips R.W., Daoud El Tannir M. (1958). Tarnish of certain dental alloys. J. Dent. Res..

[69] Fusayama T., Katayori T., Nomoto S. (1963). Corrosion of gold and amalgam placed in contact with each other. J. Dent. Res..

[70] Nomura D., Saito M., Takahashi Y., Takahashi Y., Takakura Y., Nishikawa M. (2018). Development of Orally-deliverable DNA Hydrogel by Microemulsification and Chitosan Coating. Int. J. Pharm..

[71] Grande R., Carvalho A.J. (2011). Compatible ternary blends of chitosan/poly (vinyl alcohol)/poly (lactic acid) produced by oil-in-water emulsion processing. Biomacromolecules.

[72] do Prado R.F., de Oliveira F.S., Nascimento R.D., de Vasconcellos L.M.R., Carvalho Y.R., Cairo C.A.A. (2015). Osteoblast response to porous titanium and biomimetic surface: In vitro analysis. Mat. Sci. Eng. C-Mater..

[73] De Oliveira P.T., Nanci A. (2004). Nanotexturing of titanium-based surfaces upregulates expression of bone sialoprotein and osteopontin by cultured osteogenic cells. Biomaterials.

